# Female Students at the Pennsylvania Hospital

**Published:** 1882-02

**Authors:** 


### FEMALE STUDENTS AT THE PENNSYLVANIA HOSPITAL.

An interesting contest stretching over a quarter of a century, and involving an important principle, has recently been decided in this city.

The medical and surgical clinics at the Pennsylvania Hospital are now attended by a score or more of women students from the senior class of the Women's Medical College, and students from that institution pursuing a post-graduate course. This is the first time that women students have been admitted to the general clinics at that institution, and also the first time in three years that they have attended any clinics in the hospital. Nearly a quarter of a century ago an effort was made to have the clinics opened to women students, but public opinion had not then been educated to such an innovation, and the application was negatived.

By 1873, the popular prejudice against women physicians had greatly lessened, and the managers allowed women students to attend a special clinic, which was held on Tuesdays, for their instruction. This privilege was exercised for five years, when the women ceased to attend, partly because they were not warmly encouraged by the demonstrators, on whom was imposed an additional lecture per week, and partly because they imagined that the instruction received was inferior to that given at the Wednesday and Saturday clinics for male students. At that time, the demonstrators objected to lecture before mixed classes.

A short time ago the managers of the Women's Medical College received intimation that no objections would be made to women students attending mixed clinics at the Pennsylvania Hospital, as the managers had no rule positively prohibiting it. Accordingly six young women appeared at the next clinic, and the demonstrator proceeded with the lecture as if no women were present. At the following clinic, there were a dozen women present, and at present the number of women attending is over twenty. No preference is shown them, nor does the demonstrator use false modesty in dealing with difficult subjects. The physicians in charge look upon the students of medicine only and do not distinguish between men and women. A few days ago some young women were late in arriving at Dr. Morton's clinic and found the front seats occupied. One of their number passed a note to the Doctor, requesting him to ask the gentlemen to move back, that the women might have seats. Dr. Morton replied that he did not recognize the right of women to the most desirable location in the lecture room, and that they must not expect any favors at the clinics. Dr. Morton was entirely right in this action, and such a request was wholly out of place.

